# Synthesis and Antibacterial Evaluation of Some Novel Imidazole and Benzimidazole Sulfonamides

**DOI:** 10.3390/molecules181011978

**Published:** 2013-09-26

**Authors:** Nassir N. Al-Mohammed, Yatimah Alias, Zanariah Abdullah, Raied M. Shakir, Ekhlass M. Taha, Aidil Abdul Hamid

**Affiliations:** 1 Chemistry Department, Faculty of Science, University of Malaya, Kuala Lumpur 50603, Malaysia; 2 Section for Co-curricular Courses, External Faculty Electives and TITAS (SKET), University of Malaya, Kuala Lumpur 50603, Malaysia; 3 Department of Chemistry, Collage of Science for Women, Baghdad University, Aljadriya 10071, Baghdad, Iraq; 4 School of Biosciences and Biotechnology, Faculty of Science and Technology, University Kebangsaan Malaysia, Bangi 43600, Selangor, Malaysia

**Keywords:** sulfonamide, 4-methylbenzenesulfonamide, 4-nitrobenzenesulfonamide, 4-methoxybenzenesulfonamide, imidazole, benzimidazole, antibacterial activity

## Abstract

Several new substituted sulfonamide compounds were synthesized and their structures were confirmed by ^1^H-NMR, ^13^C-NMR, FT-IR, and mass spectroscopy. The antibacterial activities of the synthesized compounds were screened against standard strains of six Gram positive and four Gram negative bacteria using the microbroth dilution assay. Most of the compounds studied showed promising activities against both types of bacteria.

## 1. Introduction

Heterocycles containing sulfonamido moieties have attracted obvious attention due to their significant biological properties and their role as pharmacophores [1,2,3,4,5,6]. Studies have shown that sulfonamide compounds were used as antibacterial agents [7,8,9], anticancer [10,11,12], anti-inflammatory, analgesic agents [13,14,15], antifungal agents [9,16] and antiviral agents [17]. Imidazole and its derivatives have been reported to be bioactive molecules in many important biological systems with a wide range of pharmacological activity. In general, they are well known as proton donors and/or acceptors in enzymatic systems, coordination system ligands and as the basis of charge–transfer processes [18,19], as well as antibacterial [20,21,22], anti-parasitic [23], antiepileptic [24], anti-inflammatory and anticancer agents [25,26,27].

In our study, new promising bioactive compounds based on the sulfonamide moiety were designed and synthesized by a simple and efficient method, followed by the evaluation of their biological activities. The synthesis emphasizes a strategy that combines two or more pharmacologically compatible moieties in one molecule by attaching a sulfonamide moiety to an imidazole, benzimidazole or another sulfonamide moiety. We believe this route has a wide range of applications and we have high expectations for the future development of new compounds.

## 2. Results and Discussion

### 2.1. Synthesis

**Scheme 1:**
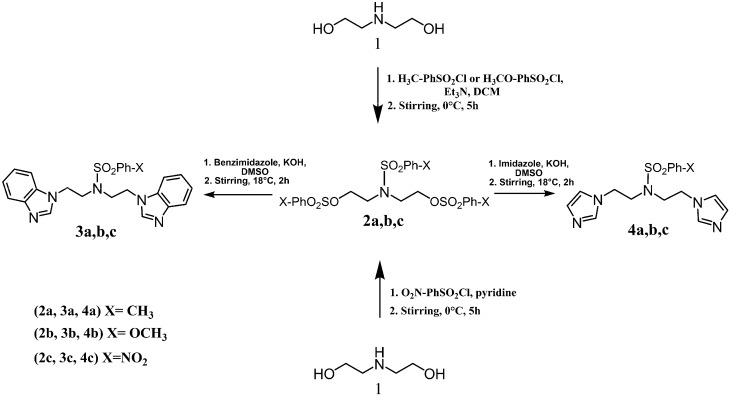
Synthesis of bis-benzimidazole sulfonamides and bis-imidazole sulfonamides **3a**–**c**, **4a**–**c**.

Bis-benzimidazole and bis-imidazole sulfonamides were synthesized from the diol **1** as shown in Scheme 1.

Three bis-benzimidazole sulfonamides and three bis-imidazole sulfonamides compounds were obtained by treating imidazole (or benzimidazole) with tris-(4-substituted benzensulfonate)- diethanolamine under basic conditions to form the corresponding bis-imidazole (or bis-benzimidazole) sulfonamides. The reaction of tris-(4-substituted benzensulfonate) with either imidazole or benzimidazole has produced symmetrical products and it is in agreement with literature [28] to synthesize tris-(4-substituted benzensulfonate)-diethanolamine. In the current study, this intermediate was applied as a reagent to synthesize symmetric bis-imidazole (or bis-benzimidazole) sulfonamide compounds. In this reaction proton abstraction from the nitrogen of imidazoles rings by potassium hydroxide [29] is considered the key step. The resulting imidazolide (or benzimidazolide) anions attack the carbon bearing the 4-substituted benzensulfonate in both sides of diethanolamine with the nitrogen atom being protected via the third 4-substituted benzenesulfonyl group.

The FTIR spectra for compounds **3a**–**c** and **4a**–**c** showed absorption bands at 1351–1375 cm^−1^ and 1150–1185 cm^−1^ which were assigned to the O=S=O group. The same compounds showed stretching absorption bands at 3100–3047 cm^−1^, 2975–2855 cm^−1^, 1590–1457 cm^−1^, and 1666–1584 cm^−1^ attributed to (C-H)_Aromatic_, (C-H)_Aliphatic_, (C=C)_Aromatic_, and (C=N), respectively. The target compounds **3b** and **4b** showed characteristic stretching absorption bands at 1,220 cm^−1^ and 1,238 cm^−1^ which were assigned to C-O-C, while the bands at 1529–1520 cm^−1^ and 1355–1340 cm^−1^ for compounds **3c** and **4c** were assigned to Ar-NO_2_.

The ^1^H-NMR spectra of compounds **3a**, **4a**, **3b**, and **4b** showed singlets at δ 2.33 ppm and δ 2.80 ppm which were assigned to the 4-methyl and 4-methoxy protons of the arylsulfonyl groups, respectively. A triplet recorded at δ 3.31–3.54 ppm and δ 3.95–4.27 ppm was assigned to the two methylene groups protons of compounds **3a**–**c** and **4a**–**c**. The aromatic protons of compounds **3a**–**c** and **4a**–**c** were recorded as multiplets in the δ 6.89–7.83 ppm range. A singlet which was observed at δ 7.42–8.16 ppm corresponds to the isolated C-H of the imidazole and benzimidazole rings. The ^13^C-NMR spectra of compounds **3a**–**c** and **4a**–**c** showed characteristic peaks in the δ 163.23–163.34 ppm, δ 149.03–153.12 ppm and δ 129.78–144.28 ppm ranges which were assigned to C_Ar_-O, C_Ar_-NO_2_ and C_Ar_-S, respectively. The peaks recorded at δ 43.09–45.77 ppm, δ 48.20–50.37 ppm and δ 56.18–56.24 ppm were attributed to the methylene and methoxy carbon atoms, correspondingly.

**Scheme 2:**
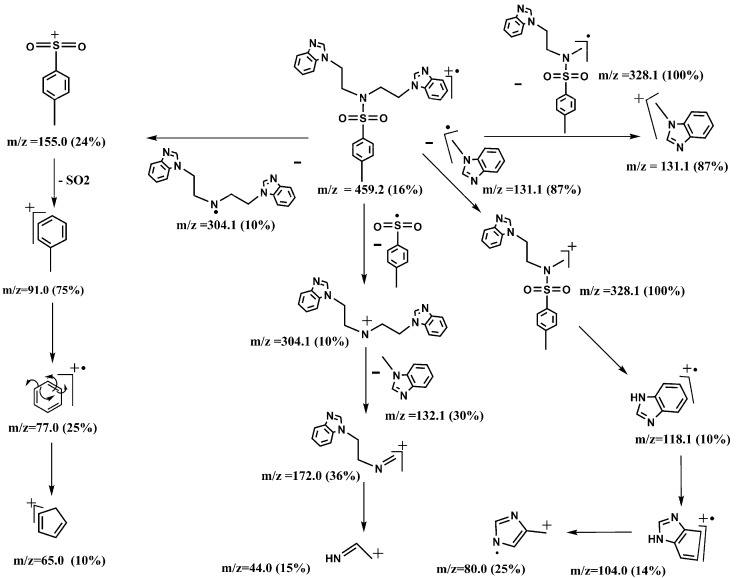
Mass fragmentation pattern of **3a**.

**Scheme 3:**
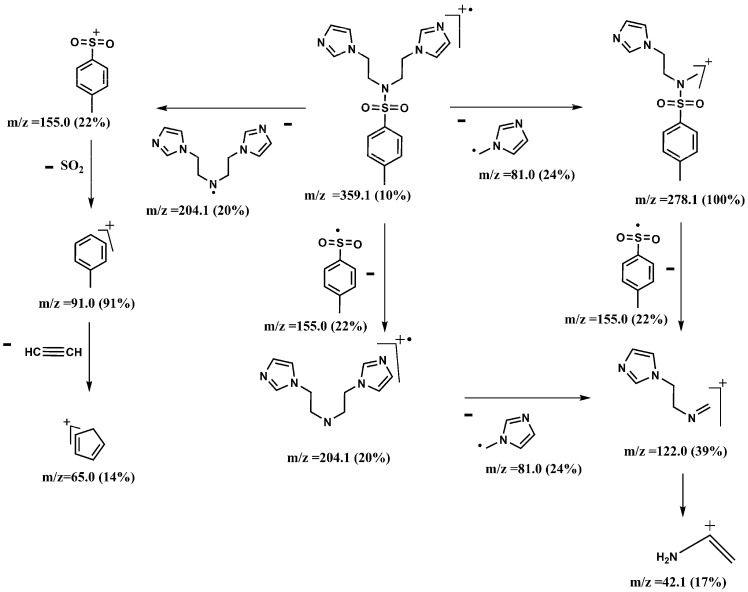
Mass fragmentation pattern of **4a**.

The mass spectra of compounds **3a** and **4a** showed various characteristic peaks. Those at *m/z* 459.2 and 359.1 were assigned to the molecular ions of **3a** and **4a**, respectively. The base peak of **3a** at *m/z* 328.1 was assigned to the *N*-(benzimidazol-1-yl)ethyl-*N*-4-dimethylbenzenesulfonamido radical, while the base peak of **4a** at *m/z* 278.1 was assigned to the *N*-(imidazole-1-yl)ethyl-(4-methylbenzene) sulfonamide-methyliumyl ion. The characteristic peaks at *m/z* 155.0 and 91.0 for both **3a** and **4a** were due to (4-methylphenyl)dioxosulfanium and 4-methylbenzene-1-ylium ions, respectively Scheme 2 and Scheme 3 show the fragmentation patterns for **3a** and **4a**, respectively.

**Scheme 4:**
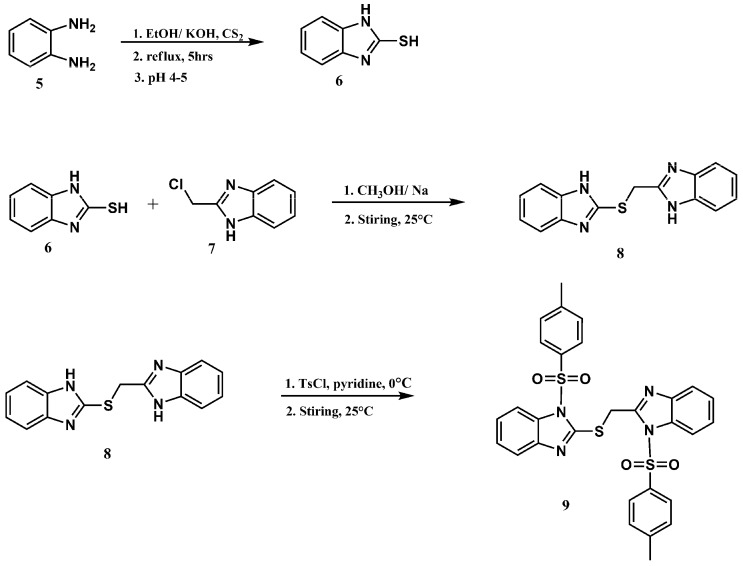
Synthesis of *N*-4-methylbenzenesulfonyl ((*N*-(4-methylbenzenesulfonyl)-benzimida zol-2-yl)methylthio)-benzimidazole (**9**).

Scheme 4 shows the preparation of compounds **9** from 2-((benzimidazol-2-yl)methylthio)-benzimidazole. A simple method was adopted to synthesize a pure heterocyclic product in good yield. 2-Mercaptobenzimidazole and sodium methoxide were stirred with 2-chloromethylbenzimidazole. A pale-yellow solid precipitated instantly due to the reactivity of -SH group then it was treated with tosyl chloride in pyridine.

The infrared spectrum of compound **8** indicated the absence of a free -SH absorption band and the appearance of S-C stretching at 748 cm^−1^, while the absorption bands at 1,365 and 1,170 cm^−1^ were assigned to the O=S=O group. The ^1^H-NMR spectrum of compound **9** showed two singlet peaks at δ 2.31, and 2.37 ppm integrating for six protons for the two methyl protons of the sulfonamido moieties. The protons of the methylene group appeared as a singlet at δ 5.18 ppm, whereas the aromatic protons appeared as multiplets and doublet peaks in the δ 7.19–7.98 ppm range. The ^13^C-NMR of compound **9** showed peaks at δ 21.77 and 21.80 ppm which was assigned to two non-corresponding methyls for two tosyl groups. The CH_2_-S carbon atom was observed at δ 31.20 ppm.

**Scheme 5:**
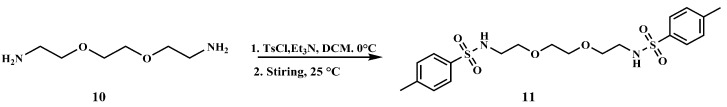
Synthesis of 4-methyl-*N*-(2-{2-[2-(4-methylbenzenesulfonamido)ethoxy]ethoxy} ethyl)-benzenesulfonamide (**11)**.

Scheme 5 shows the preparation of compound **11** from 2,2-(ethylenedioxy)bis(ethylamine) by treating with tosyl chloride and triethylamine in dry dichloromethane that produced a significant yield of pure bis-sulfonamide compound **11**.

The FTIR spectrum for compound **11** showed absorption bands at 3276 cm^−1^ indicating the presence of a N-H group, 1088 cm^−1^ for a C-O-C group, 1124 cm^−1^ for C-C-O vibrations, and the peaks at 1317, 1152 cm^-1^ were assigned to the O=S=O group. The ^1^H-NMR spectrum of compound **11** showed characteristic doublet peaks at δ 7.27 ppm and 7.73 ppm which were assigned to the aromatic protons. The methylene groups were recorded as a quartet at δ 3.09 ppm and a triplet at δ 3.50 ppm integrating for eight and four protons, respectively. The amino protons of compound **11** appeared as a triplet at δ 5.50 ppm. The corresponding methyls for two tosyl groups were observed as a single peak at δ 2.39 ppm integrating for six protons. The ^13^C-NMR of compound **11** showed aromatic carbon peaks at δ 143.45, 137.07, 129.78 and 127.18 ppm. The peak at δ 21.59 ppm was assigned to the two corresponding methyl groups while, peaks of 4 × CH_2_-O were observed at δ 69.78 and 70.43 ppm. The structures of compounds **3a**, **4a**, **9** and **11** were further characterized by single crystal X-ray diffraction which indicated tetragonal and triclinic crystal systems for **3a** and **4a**, respectively, while the crystal structures of **9** and **11** have been reported [30,31]. Crystallographic data for compounds **3a** and **4a** have been deposited with the Cambridge Crystallographic Data Centre as supplementary publication numbers CCDC 923664 and CCDC 923665, respectively. Copies of the data can be obtained free of charge via http://www.ccdc.cam.ac.uk/conts/retrieving.html, or from the Cambridge Crystallographic Data Centre, 12 Union Road, Cambridge CB21EZ, UK; fax: (+44)-1223-336-033; or [e-mail: deposit@ccdc.cam.ac.uk].

### 2.2. Antibacterial Activities

**Figure 1:**
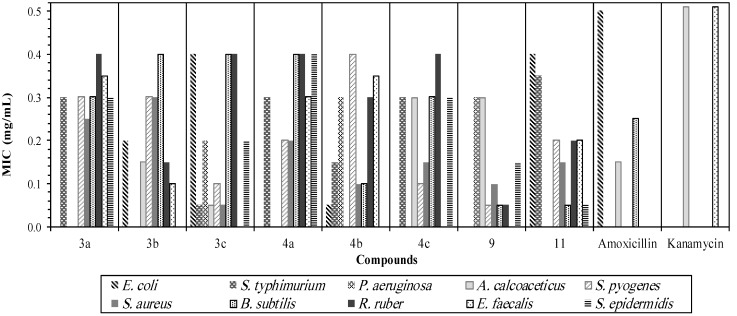
MIC’s Histogram for synthesized compounds (0.05–0.40 mg/mL concentration) versus ten strains of bacteria.

In an *in vitro* antibacterial bioassay, the eight compounds **3a**–**c**, **4a**–**c**, **9** and **11** were evaluated by microbroth dilution assays using representative standard strains of Gram-positive and Gram-negative bacteria, and the results are listed in Table 1. Minimum inhibitory concentrations (mg/mL) of the compounds against the test microorganisms were determined. It was shown (Figure 1) that the majority of the compounds studied possessed significant antibacterial activity towards most of the selected microorganisms. The highest activities were observed for compounds **9** and **11**, followed by **3c** and **4c** then **3b** and **4b**. Compounds **3a** and **4a** showed the least antibacterial activity for the selected concentration range, as shown in Figure 1. In the structure-activity relationships (SAR) studies, it has been reported that the incorporation of two different pharamacophores in a single structure enhanced the resulting compounds’ biological activities [32,33,34]. The presence of substituents on aromatic rings also affects the antibacterial activities of compounds. Compounds with resonance electron-withdrawing substitution (nitro) showed greater antibacterial activities than those with electron-donating substituent groups (methyl and methoxy) [32,33,34] and this is clearly shown in the case of compounds **3a**–**c** and **4a**–**c**. Studies have also shown that the presence of a sulfur atom as a sulfide in drugs provides a greater stability to the three dimensional structure of the molecule [35]. It is observed that the presence of sulfur in compound **9** has a significant contribution to the antibacterial activities against Gram positive and Gram negative bacteria. This is believed to due to the existence of a toxophoric (-N=C-S-) group [36,37,38]. Furthermore, the attachment of two toxophoric groups (amine) with benzensulfonyl moieties in compound **11** enhanced the antibacterial activity against most of both kinds of bacteria [39,40,41].

It is obvious from the overall antibacterial results that different compounds reacted in different ways against bacteria. In these compounds, strains of Gram-positive bacteria seem to be more sensitive than Gram negative micro-organisms.

**Table 1:** Antibacterial activities of compounds studied.

No.	Structure of samples	Bacteria/MICs (mg/mL)									
		*Gram-negative bacteria*				*Gram-positive bacteria*					
		*Escherichia coli*	*Salmonella typhimurium*	*Pseudomonas aeruginosa*	*Acinetobacter calcoaceticus*	*Streptococcus pyogenes*	*Staphylococcus aureus*	*Bacillus subtilis*	*Rodococcus ruber*	*Enterococcus faecalis*	*Staphylococcus epidermidis*
**3a**	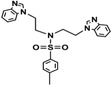	**>0.5**	**0.30**	**>0.5**	**>0.5**	**0.30**	**0.25**	**0.30**	**0.40**	**0.35**	**0.30**
**3b**	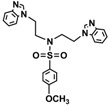	**0.2**	**>0.5**	**>0.5**	**0.15**	**0.30**	**0.30**	**0.40**	**0.15**	**0.10**	**>0.5**
**3c**	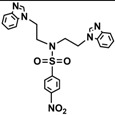	**0.40**	**0.05**	**0.20**	**0.05**	**0.10**	**0.05**	**0.40**	**0.40**	**>0.5**	**0.20**
**4a**	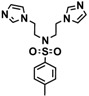	**>0.5**	**0.30**	**>0.5**	**>0.5**	**0.20**	**0.20**	**0.40**	**0.40**	**0.30**	**0.40**
**4b**	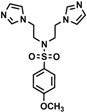	**0.05**	**0.15**	**0.30**	**>0.5**	**0.40**	**0.10**	**0.10**	**0.30**	**0.35**	**>0.5**
**4c**	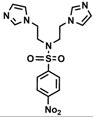	**>0.5**	**0.30**	**>0.5**	**0.30**	**0.10**	**0.15**	**0.30**	**0.40**	**>0.5**	**0.30**
**9**	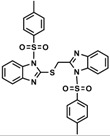	**>0.5**	**>0.5**	**0.30**	**0.30**	**0.05**	**0.10**	**0.05**	**0.05**	**>0.5**	**0.15**
**11**	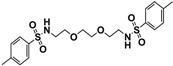	**0.40**	**0.35**	**>0.5**	**>0.5**	**0.20**	**0.15**	**0.05**	**0.20**	**0.20**	**0.05**
**AM**	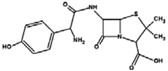	**<0.05**	**<0.05**	**Nd**	**0.15**	**0.05**	**<0.05**	**0.25**	**<0.05**	**<0.05**	**nd**
**KA**	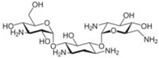	**<0.05**	**<0.05**	**<0.05**	**>0.5**	**<0.05**	**<0.05**	**<0.05**	**<0.05**	**>0.5**	**<0.05**

**MIC**: Minimum inhibitory concentration, AM: Amoxicillin, KA: Kanamycin, nd: not detected.

Thus, comparing the results of both the synthesized compounds and amoxicillin against a *β-*lactam resistant Gram-positive bacteria (*Staphylococcus epidermidis)* demonstrated interesting antibacterial inhibitory values for most of the synthesized compounds. The MIC values are between 0.05 mg/mL (for compound **11**) to 0.4 mg/mL (for compound **4a**) whereas the *β-*lactam antibiotic amoxicillin was inactive against this strain of Gram-positive bacteria.

In addition, compounds **3c**, **4b**, and **9** showed significant activities, with MIC values (0.2, 0.3 and 0.3 mg/mL, respectively) toward a *β-*lactam resistant Gram-negative bacterium (*Pseudomonas* *aeruginosa*) when compared to the antibiotic amoxicillin. Compounds **4b**, **9** and **11** with MIC values of 0.10, 0.05 and 0.05 mg/mL, respectively showed interesting antibacterial activities against *Bacillus subtilis*, which required a high dose of amoxicillin (0.25 mg/mL) [42]*.* Compounds **3a**–**b**, **4a**,**b**, and **11** demonstrated inhibitory effects ranging between 0.1–0.35 mg/mL against the Gram-positive bacterium *Enterococcus faecalis*, however, the antibiotic kanamycin was inactive toward the samples at the concentration range of this study (0.05–0.5 mg/mL). Both commercial antibiotics amoxicillin and kanamycin exhibited MIC values (0.15 mg/mL and >0.5 mg/mL, sequentially) against *Acinetobacter calcoaceticus*, while compound **3c** exhibited a significant antibacterial inhibitory effect at 0.05 mg/mL against the mentioned Gram-negative bacteria.

## 3. Experimental

### 3.1. General

The IR spectra were obtained with a Perkin Elmer 400 Fourier Transform Infrared (FTIR) spectrometer. ^1^H and ^13^C-NMR spectra were recorded on Jeol Lambda and ECA DELTA spectrometers at 400 MHz. The mass spectra were recorded using an Agilent 5975 system for EI/MS and a Finnigan TSQ7000 for HREI/MS (NUS, Singapore). Melting points were measured on a Gallenkamp melting point apparatus in open-end capillary tubes and are uncorrected. Thin layer chromatography was carried out on pre-coated silica gel plates (0.25 mm, 20 × 20 cm, 60F_254_, E. Merck). Flash column chromatography on silica gel 60 (230–400 mesh, E. Merck).General grade solvents and reagents were purchased from commercial suppliers and used without further purification.

### 3.2. Synthesis N-(4-Methylbenzenesulfonyl)-bis((4-methylbenzenesulfonyl(oxy))ethyl)amine (**2a**) and N-(4-Methoxybenzenesulfonyl)-bis((4-methoxybenzenesulfonyl(oxy))-ethyl)amine (**2b**)

These compounds were prepared according to the modified procedure described in [28]. Diethanolamine (5.5 g, 0.0524 mol) was dissolved in distilled dichloromethane (100 mL). The solution was cooled to 0 °C and then triethylamine (24.4 mL, 17.78 g, 0.176 mol) was added. With the temperature maintained at 0 °C, solid *p*-toluenesulfonyl chloride (31.4 g, 0.164 mol) or (4-methoxybenzenesulfonyl chloride (34.1 g, 0.165 mol) were added in portions with vigorous stirring over the course of 5 h to obtain compounds **2a** or **2b**, respectively. The reaction mixture was stirred at room temperature overnight. A pale yellow filtrate was produced from Et_3_NHCl filtration, washed three times with 1 mol/L HCl, followed by 5 × 40 mL portions of water and 5 × 40 mL portions of saturated NaHCO_3_ solution. The organic layer was dried over anhydrous magnesium sulfate and evaporated to obtain yellow viscous liquid that solidified after 5–7 days.

*N-(4-Methylbenzenesulfonyl)-bis((4-methylbenzenesulfonyl(oxy))ethyl)amine* (**2a**)***.*** White solid; Yield: (87%) ; m.p. 98–100 °C; FTIR (cm^−1^): 3098, 3045 (C-H)_Ar_, 2967 (C-H)_Aliph_, 1498, 1472 (C=C)_Ar_, 1360, 1155 (O=S=O); ^1^H-NMR (CDCl_3_) δ ppm: 7.77–7.65 (m, 6H, Ar-H), 7.54–7.48 (m, 4H, Ar-H ), 7.44–7.40 (m, 2H, Ar-H), 4.45 (t, *J* = 6.23 Hz, 4H, CH_2_-OTs), 3.23 (t, *J* = 6.23 Hz, 4H, CH_2_-N-Ts), 2.33 (two singlets, 9H, Ar-CH_3_). ^13^C-NMR (CDCl_3_) δ ppm: 144.60 (2 × C_Ar_-S-O), 142.92 (C_Ar_-S-N), 140.22 (2 × C_Ar_-CH_3_), 138.55 (C_Ar_-CH_3_), 131.04 (4 × CH_Ar_), 129.40 (2 × CH_Ar_), 127.23 (4 × CH_Ar_), 127.10 (2 × CH_Ar_), 55.79 (2 × CH_2_-OTs), 46.66 (2 × CH_2_-N-Ts), 22.30 (2 × CH_3_-Ar), 20.52 (CH_3_-Ar).

*N-(4-Methoxybenzenesulfonyl)-bis((4-methoxybenzenesulfonyl(oxy))ethyl)amine* (**2b**) Off-white solid; Yield: 92%; m.p 157–158 °C; FTIR (cm^−1^): 3096, 3050 (C-H)_Ar_, 2922 (C-H)_Aliph_, 1494, 1466 (C=C)_Ar_, 1366, 1160 (O=S=O), 1220 (C-O-C); ^1^H-NMR (DMSO-*d*_6_) δ ppm: 7.80–7.76 (m, 4H, Ar-H), 7.65–7.51 (m, 2H, Ar-H), 7.19–7.16 (m, 4H, Ar-H), 7.06–7.04 (m, 2H, Ar-H), 3.98 (t, *J* = 5.77 Hz, 4H, CH_2_-OTs), 3.28 (t, *J* = 5.77 Hz, 4H, CH_2_-N-Ts), 3.85 (two singlets, 9H, Ar-O-CH_3_); ^13^C-NMR (DMSO-*d*_6_) δ ppm: 164.18 (2 × C_Ar_-OCH_3_), 163.30 (C_Ar_-OCH_3_), 130.48 (4 × CH_Ar_), 129.94 (C_Ar_-S-O), 129.73 (2 × CH_Ar_), 127.58 (2 × CH_Ar_), 126.62 (2 × C_Ar_-S-O), 115.50 (4 × CH_Ar_), 115.13 (2 × CH_Ar_), 68.51 (2 × CH_2_-OTs), 56.41, 56.25 (3 × OCH_3_), 47.80 (2 × CH_2_-N-Ts).

### 3.3. Synthesis N-(4-Nitrobenzenesulfonyl)-bis((4-nitrobenzenesulfonyl(oxy))ethyl)amine (**2c**)

A solution of 4-nitrobenzenesulfonyl chloride (21 g, 0.095 mol) in pyridine (40 mL) was added dropwise to a solution of diethanolamine (3.5 g, 0.03 mol) in pyridine (10 mL) while maintaining the temperature at 0 °C. The mixture was stirred at room temperature overnight and then poured into a beaker containing 400 mL of ice water. The mixture was then stirred for another 30 minutes, extracted with dichloromethane and washed with distilled water (3 × 50 mL). The organic layer was dried with anhydrous magnesium sulfate and the solvent evaporated under reduced pressure to give a green viscous liquid which solidified after five days to give a greenish-brown solid; Yield: 57%; m.p 184–186 °C; FTIR (cm^−1^): 3095, 3048 (C-H)_Ar_, 2950 (C-H)_Aliph_, 1518, 1476 (C=C)_Ar_, 1360, 1174 (O=S=O), 1532, 1347 (Ar-NO_2_); ^1^H-NMR (DMSO-*d*_6_) δ ppm: 8.43–8.39 (m, 4H, Ar-H), 8.35–8.32 (m, 2H, Ar-H), 8.14–7.11 (m, 6H, Ar-H), 3.55 (t, *J* = 6.25 Hz, 4H, CH_2_-OTs), 3.32 (t, *J* = 6.25 Hz, 4H, CH_2_-N-Ts); ^13^C-NMR (DMSO-*d*_6_) δ ppm: 159.20 (2 × C_Ar_-NO_2_), 158.78 (2 × C_Ar_-S-O), 149.08 (CH_Ar_-NO_2_), 141.23 (C_Ar_-S-O), 130.88 (4 × CH_Ar_), 127.30 (2 × CH_Ar_), 125.43 (4 × CH_Ar_), 123.84 (2 × CH_Ar_), 63.53 (2 × CH_2_-OTs), 53.44 (2 × CH_2_-N-Ts).

### 3.4. General Procedure for Synthesis of **3a**, **3b**, **3c** and **4a**, **4b**, **4c**

Potassium hydroxide (1.85 g, 0.033 mol) was added to a solution of imidazole or benzimidazole (0.022 mol) in DMSO (20 mL) and the mixture was stirred for 30 min at 20 °C, and the corresponding **2a**, **2b** or **2c** (0.01 mol; 5.67 g, 6.15 g and 6.60 g respectively) was added portionwise under vigorous stirring in a water bath. The stirring was continued for another 2 h, the water (200 mL) was then added to the mixture which was extracted with chloroform (6 × 25 mL). The combined extracts were washed with water and dried over anhydrous magnesium sulfate. The solvent was evaporated off and the product was recrystallized from methanol.

*N,N-bis[(Benzimidazol-1-yl)ethyl]-4-methylbenzenesulfonamide* (**3a**). White solid; Yield: 94%; a single crystal was obtained properly for X-ray structural determination by using DMF; m.p.192–194 °C; FTIR (cm^−1^): 3098, 3047 (C-H)_Ar_, 2927 (C-H)_Aliph_, 1666, 1615, 1598 (C=N)_Ar_, 1498, 1460 (C=C)_Ar_, 1362, 1150 (O=S=O); ^1^H-NMR (DMSO-*d_6_*) δ ppm: 8.09 (s, 2H, C-H_BImidazole_), 7.64–7.60 (m, 4 H, 2 × C-H_Ar_, 2 × C-H_BImidazole_), 7.44 (d, 2H, *J* = 8.15 Hz, C-H_Ar_), 7.30–7.19 (m, 6H, C-H_BImidazole_), 4.27 (t, *J* = 6.80 Hz, 4H, 2 × CH_2_-N_Ar_), 3.49 (t, *J* = 6.80 Hz, 4H, 2 × CH_2_-N), 2.33 (s, 3H, -CH_3_); ^13^C-NMR (DMSO-*d*_6_) δ ppm: 144.57 (2 × CH_BImidazole_), 144.20 (C_Ar_-S), 143.84 (2 × C_BImidazole_), 135.36 (C_Ar_-CH_3_), 134.04 (2 × C_BImidazole_), 130.43 (2 × CH_Ar_), 127.49 (2 × CH_BImidazole_), 123.07 (2 × CH_BImidazole_), 122.15 (2 × CH_Ar_), 120.06 (2 × CH_BImidazole_), 110.59 (2 × CH_BImidazole_), 48.71 (2 × CH_2_-N), 43.77 (2 × CH_2_-N_Ar_), 21.51 (CH_3_); EIMS (*m/z*): 459 (16%, M^+^), 278 (100%), 304 (10%), 278 (13%), 172 (36%), 155 (24%), 131 (87%), 91 (75%).

*N,N-bis[2-(Benzimidazol-1-yl)ethyl]-4-methoxybenzenesulfonamide* (**3b**). White solid; Yield: 84%; m.p. 138–140 °C; FTIR (cm^−1^): 3096, 3055 (C-H)_Ar_, 2910 (C-H)_Aliph_, 1666, 1615, 1597 (C=N)_Ar_, 1494, 1460 (C=C)_Ar_, 1375, 1164 (O=S=O), 1220 (C-O-C); ^1^H-NMR (DMSO-*d*_6_) δ ppm: 8.09 (s, 2H, C-H_BImidazole_), 7.68–7.62 (m, 4H, 2 × C-H_Ar_, 2 × C-H_BImidazole_), 7.45 (d, 2H, *J* = 8.15 Hz, C-H_Ar_), 7.28–7.15 (m, 4H, C-H_BImidazole_), 7.00–6.96 (m, 2H, C-H_BImidazole_), 4.27 (t, *J* = 6.80 Hz, 4H, 2 × CH_2_-N), 3.80 (s, 3H, OCH_3_), 3.48 (t, *J* = 6.80 Hz, 4H, 2 × CH_2_-N); ^13^C-NMR (DMSO-*d*_6_) δppm 163.23 (C_Ar_-O), 144.51 (2 × CH_BImidazole_), 143.83 (2 × C_BImidazole_), 134.07 (2 × C_BImidazole_), 129.78 (C_Ar_-S), 129.70 (2 × CH_Ar_), 123.05 (2 × CH_BImidazole_), 122.19 (2 × CH_BImidazole_), 120.08 (2 × CH_BImidazole_), 115.10 (2 × CH_Ar_), 110.60 (2 × CH_BImidazole_), 56.18 (O-CH_3_), 48.66 (2 × CH_2_-N), 43.75 (2 × CH_2_-N); EIMS (*m/z*): 475 (20%, M^+^), 344 (100%), 304 (10%), 278 (13%), 172 (44%), 107 (35%), 131(75%), 91(80%).

*N,N-bis[(Benzimidazol-1-yl)ethyl]-4-nitrobenzenesulfonamide* (**3c**). Pale green solid; Yield: 62%; m.p. 80–82 °C; FTIR (cm^−1^): 3102, 3055 (C-H)_Ar_, 2950 (C-H)_Aliph_, 1660, 1615, 1598 (C=N)_Ar_, 1510, 1485 (C=C)_Ar_, 1360, 1178 (O=S=O), 1529, 1340 (Ar-NO_2_); ^1^H-NMR (DMSO-*d*_6_) δ ppm: 8.16 (s, 2H, C-H_BImidazole_), 7.78–7.73 (m, 4H, 2 × C-H_Ar_, 2 × C-H_BImidazole_), 7.52 (d, 2H, *J* = 8.15 Hz, 2 × C-H_Ar_), 7.31–7.22 (m, 4H,C-H_BImidazole_), 6.99–6.94 (m, 2H, C-H_BImidazole_), 4.23 (t, *J* = 6.80 Hz, 4H, 2 × CH_2_-N), 3.54 (t, *J* = 6.80 Hz, 4H, 2 × CH_2_-N); ^13^C-NMR (DMSO-*d*_6_) δ ppm: 153.12 (C_Ar_-NO_2_), 145.57 (2 × CH_BImidazole_), 143.65 (2 × C_BImidazole_), 134.12 (2 × C_BImidazole_), 130.04 (C_Ar_-S), 128.44 (2 × CH_Ar_), 124.95 (2 × CH_BImidazole_), 120.34 (2 × CH_BImidazole_), 118.88 (2 × CH_BImidazole_), 117.10 (2 × CH), 111.07 (2 × CH_BImidazole_), 50.37 (2 × CH_2_-N), 44.75 (2 × CH_2_-N); EIMS (*m/z*): 490 (16%, M^+^), 359 (100%), 304 (20%), 278 (30%), 186 (45%), 22 (75%).

*N,N-bis[(Imidazol-1-yl)ethyl]-4-methylbenzenesulfonamide* (**4a**). White solid; Yield: 82%; a single crystal was obtained for X-ray analysis by using acetonitrile; m.p. 92–94 °C; FTIR (cm^−1^) 3093 (C-H)_Ar_, 2975 (C-H)_Aliph_, 1597 (C=N)_Ar_, 1510, 1457 (C=C)_Ar_, 1351, 1159 (O=S=O); ^1^H-NMR (DMSO-*d_6_*) δ ppm: 7.72 (d, *J* = 8.15 Hz, 2 × C-H_Ar_), 7.54 (s, 2 × C-H_Imidazole_), 7.41(d, *J* = 8.15 Hz, 2 × C-H_Ar_), 7.12 (s, 2H, C-H_Imidazole_), 6.89 (s, 2H, C-H_Imidazole_), 3.96 (t, *J* = 6.80 Hz, 4H, 2 × CH_2_-N), 3.32 (t, *J* = 6.80 Hz, 4H, 2 × CH_2_-N), 2.39 (s, 3H, CH_3_); ^13^C-NMR (DMSO-*d*_6_) δ ppm 144.28 (C_Ar_-S), 137.87 (2 × CH_Imidazole_), 135.53 (C_Ar_-CH_3_), 130.49 (2 × CH_Ar_), 128.99 (2 × CH_Imidazole_), 127.56 (2 × CH_Ar_), 119.95 (2 × CH_Imidazole_), 50.30 (2 × CH_2-_N), 45.75 (2 × CH_2_-N), 21.49 (-CH_3_); EIMS (*m/z*): 359 (10%, M^+^), 278 (100%), 204 (20%), 155 (22%), 122 (39%), 91 (97%).

*N,N-bis[2-(Imidazol-1-yl)ethyl]-4-methoxybenzenesulfonamide* (**4b**). Off-white solid; Yield: 90%; m.p. 66–68 °C; FTIR (cm^−1^): 3100 (C-H)_Ar_, 2855 (C-H)_Aliph_, 1584 (C=N)_Ar_, 1530, 1460 (C=C)_Ar_, 1360, 1170 (O=S=O), 1238 (C-O-C); ^1^H-NMR (DMSO-*d_6_*) δ ppm: 7.76 (d, *J* = 8.61 Hz, 2H, C-H_Ar_), 7.55 (s, 2H, C-H_Imidazole_), 7.14–7.10 (m, 4H, 2 × C-H_Ar_, 2 × C-H_Imidazole_), 6.89 (s, 2H, C-H_Imidazole_), 3.97 (t, *J* = 6.80 Hz, 4H, 2 × CH_2_-N), 3.82 (s, 3H, O-CH_3_), 3.31 (t, *J* = 6.80 Hz, 4H, 2 × CH_2_-N); ^13^C-NMR (DMSO-*d*_6_) δ ppm: 163.34 (C_Ar_-O), 137.92 (2 × CH_Imidazole_), 129.95 (2 × CH_Imidazole_), 129.86 (C_Ar_-S), 129.01 (2 × CH_Ar_), 120.06 (2 × CH_Imidazole_), 115.23 (2 × CH_Ar_), 56.24 (O-CH_3_), 50.24 (2 × CH_2_-N), 45.67 (2 × CH_2_-N); EIMS (*m/z*): 375 (22% , M^+^), 294 (100% ), 204 (20%), 171 (36%), 122 (35%), 107 (35%).

*N,N-bis[(Imidazol-1-yl)ethyl]-4-nitrobenzenesulfonamide* (**4c**). Pale green solid; Yield: 54%; m.p. 46–48 °C; FTIR (cm^−1^): 3080 (C-H)_Ar_, 2890 (C-H)_Aliph_, 1620 (C=N)_Ar_, 1574, 1460 (C=C)_Ar_, 1375, 1185 (O=S=O), 1520, 1355 (Ar-NO_2_); ^1^H-NMR (DMSO-*d_6_*) δ ppm 7.96 (d, *J* = 8.60 Hz, 2H, C-H_Ar_), 7.78 (s, 2H, C-H_Imidazole_), 7.36–7.32 (m, 4H, 2 × C-H_Ar_, 2 × C-H_IMi_), 6.95 (s, 2H, C-H_Imidazole_), 3.95 (t, *J* = 6.80 Hz, 4H, 2 × CH_2_-N), 3.55(t, *J* = 6.80 Hz, 4H, 2 × CH_2_-N). ^13^C-NMR (DMSO-*d*_6_) δ ppm 149.03 (C_Ar_-NO_2_), 138.23 (2 × CH_Imidazole_), 130.11 (2 × CH_Imidazole_), 129.78 (C_Ar_-S), 128.12 (2 × CH_Ar_), 122.06 (2 × CH_Imidazole_), 112.14 (2 × CH_Ar_), 48.20 (2 × CH_2_-N), 43.09 (2 × CH_2_-N); EIMS (*m/z*): 390 (20%, M^+^), 309 (60%), 204 (20%), 186 (30%), 122 (100%), 81 (75%).

### 3.5. Synthesis 2-Mercaptobenzimidazole (**6**)

Prepared according to the modified procedure in [43]. *o*-Phenylenediamine (7 g, 0.065 mol) was dissolved in absolute ethanol (40 mL) in a 250 mL flask. Carbon disulfide (10 mL) was then added to the solution followed by the addition of a solution of potassium hydroxide (4.35 g, 0.077 mol) in water (25 mL). The reaction mixture was thoroughly stirred and refluxed for 5 h. It was initially yellow, then turned to brown as the reaction progressed. Evolution of hydrogen sulfide gas was observed. After completion of the reaction, the mixture was poured into a beaker with ice-water and acidified with 4N hydrochloric acid to pH 4–5 to obtain a white precipitate. The precipitate was then filtered and recrystallized from ethanol. White solid; Yield 84%; m.p. 303–305 °C; FTIR (cm^−1^): 3,250 (N-H), 1,557 (C=C)_Ar_, 1,633 (C=O), 1,517 (C=N).

### 3.6. Synthesis 2-((Benzimidazol-2-yl)methylthio)-benzimidazole (**8**)

Prepared according to modified procedure in [44]. Sodium (0.85 g, 0.037 mol) was added to a solution of 2-mercaptobenzimidazole (5 g, 0.033 mol) in anhydrous methanol (60 mL) and the mixture was vigorously stirred for 20 minutes. 2-Chloromethylbenzimidazole (5.55 g, 0.033 mol) was added portion-wise to the mixture and left to stir for 2 h. A yellow precipitate was formed, filtered and washed with methanol, cold water and dried in an oven. The crude product was recrystallized from tetrahydrofuran to give a white solid of the title compound. White solid; Yield: 96%; m.p. 255–257 °C; FTIR (cm^−1^): 3372 (N-H), 3090 (C-H)_Ar_, 2975 (C-H)_Aliph_, 1621 (C=N)_Ar_, 1590 (C=C)_Ar_, 748 (C-S); ^1^H-NMR (DMSO-*d_6_*) δ ppm: 12.52 (s, H, N-H), 12.23 (s, H, N-H), 7.41–7.32 (m, 4H, C-H_Ar_), 6.97–6.90 (m, 4H, C-H_Ar_), 4.93 (s, 2H, CH_2_-S). ^13^C-NMR (DMSO-d_6_) δ ppm: 153.11 (C_Ar_-S), 149.73 (C_Ar_-S-CH_2_), 140.32, 139.66 (4 × C_Ar_), 125.34, 123.92 (4 × CH_Ar_), 114.53, 116.03 (4 × CH_Ar_) 33.12 (CH_2_).

### 3.7. Synthesis N-4-Methylbenzenesulfonyl ((N-(4-methylbenzenesulfonyl)benzimidazol-2-yl)methylthio)-benzimidazole (**9**)

A solution of *p*-toluenesulfonyl chloride (7.44 g, 0.018 mol) in pyridine (25 mL) was added dropwise to a solution of 2-((benzimidazol-2-yl)methylthio)-benzimidazole (**8**) (5 g, 0.018 mol) in pyridine (25 mL) at 0 °C, within 3 h. The mixture was stirred at room temperature and left overnight. It was then quenched with ice-water, stirred for another 20 minutes, extracted with dichloromethane (5 × 30 mL) and washed with distilled water. The organic layer was dried over anhydrous magnesium sulfate and solvent evaporated off. The crude product was purified by using flash chromatography with hexane-ethyl acetate (4:1, v/v) as eluent. The obtained solid was recrystallized from acetonitrile to give colorless crystals of compound (**9**). Colorless crystals; Yield 52%; m.p. 204–206 °C; FTIR (cm^−1^) 3087 (C-H)_Ar_, 2990 (C-H)_Aliph_, 1660, 1,616 (C=N)_Ar_, 1593, 1462 (C=C)_Ar_, 1365, 1170 (O=S=O), 752 (C-S); ^1^H-NMR (CDCl_3_) δ ppm 7.98–7.89 (m, 6H, C-H_Ar_), 7.64–7.49 (m, 2H, C-H_Ar_), 7.38–7.24 (m, 6H, C-H_Ar_), 7.19 (d, *J* = 8.61, 2H, C-H_Ar_), 5.18 (s, 2H, CH_2_), 2.31, 2.37 (two singlets, 6H, 2 × CH_3_); ^13^C-NMR (CDCl_3_) δ ppm 151.81 (C_BImidazole_-S), 149.43 (C_BImidazole_-CH_2_-S), 146.42, 146.18 (2 × C_Ar_-S), 143.22, 141.80 (2 × C_BImidazole_-N), 135.04, 134.57 (2 × C_Ar_-CH_3_), 133.98, 133.16 (2 × C_BImidazole_-N), 130.38, 130.15 (4 × CH_Ar_), 127.54, 127.45 (4 × CH_Ar_), 125.52, 124.91, 124.69, 124.14, 120.56, 118.95, 113.56, 112.95 (8×CH_BImidazole_), 31.20 (CH_2_-S), 21.80, 21.77 (2 × CH_3_); EIMS (*m/z*): 588 (45%, M^+^), 433 (80%), 278 (35%), 155 (85%), 148 (12%), 91 (100%).

### 3.8. Synthesis 4-Methyl-N-(2-{2-[2-(4-ethylbenzenesulfonamido)ethoxy]ethoxy}ethyl)benzenesulfon-amide (**11**)

*p*-Toluenesulfonyl chloride (5.66 g, 0.029 mol) was dissolved in dry dichloromethane (25 mL) and added dropwise to a stirring solution of 1,8-diamino-3,6-dioxaoctane(2,2′-(ethylenedioxy)bis-(ethyl amine) (2 g, 0.013 mol) and triethylamine (4.42 mL, 0.031 mol) in dichloromethane (25 mL) at 0 °C. The mixture was stirred further at room temperature for overnight and extracted with water and saturated solution of NaHCO_3_ (3 × 15 mL). The organic layer was dried over anhydrous magnesium sulfate and the solvent was evaporated off. Colorless crystals were obtained through slow evaporation of methanolic solution at room temperature. Yield: 92%; m.p. 88–90 °C; FTIR (cm^−1^) 3276 (NH), 3090 (C-H)_Ar_, 2921, 2898, 2872 (C-H)_Aliph_, 1596 (C=C)_Ar_, 1317, 1152 (O=S=O), 1088 (C-O-C), 1124 (C-C-O); ^1^H-NMR (CDCl_3_) δ ppm: 7.73 (d, *J* = 8.61 Hz, 4H, C-H_Ar_), 7.27 (d, *J* = 8.61 Hz, 4H, C-H_Ar_), 5.50 (t, *J* = 5.89 Hz, 2H, 2 × NH), 3.50 (t, *J* = 5.44 Hz, 8H, 4 × CH_2-_O), 3.09 (q, *J* = 8.15 Hz, 4H, 2 × CH_2_-NH), 2.39 (s, 6H, 2 × CH_3_); ^13^C-NMR (CDCl_3_) δ ppm: 143.45 (2 × C_Ar_-S), 137.07 (2 × C_Ar_-CH_3_), 129.78 (4 × CH_Ar_), 127.18 (4 × CH_Ar_), 70.43 (2 × CH_2_-O), 69.78 (2 × CH_2_-O), 42.99 (2 × CH_2_-N), 21.59 (2 × CH_3_); EIMS (*m/z*): 456 (25%, M^+^), 301 (100%), 155 (70%), 146 (35%), 91 (90%).

### 3.9. Antibacterial Evaluation

The antibacterial activity of synthesized compounds **3a**–**c**, **4a**–**c**, **9** and **11** was tested against standard strains of ten bacteria. They were obtained from the collection of the School of Biosciences and Biotechnology, Faculty of Science and Technology, University Kebangsaan, Malaysia. These strains included Gram positive bacteria: *Streptococcus pyogenes* ATCC19615, *Staphylococcus aureus* ATCC 29213, *Bacillus subtilis* ATCC6051, *Rodococcus Ruber* ATCC27863, *Enterococcus faecalis* ATCC 29212, *Staphylococcus epidermidis* ATCC12228 and Gram negative bacteria*: Escherichia coli* ATCC10538, *Salmonella typhimurium* ATCC14028, *Pseudomonas aeruginosa* ATCC15442, *Acinetobacter calcoaceticus* ATCC 23055.

The antibacterial activity was assessed in terms of minimum inhibitory concentrations (MICs) by using microbroth dilution assays according to the CLSI guidelines [45]. All the tested compounds were dissolved in dimethylsulfoxide (DMSO) which was used as negative control with concentrations range from 0.05 to 0.5 mg/mL. Commercial antibiotics amoxicillin and kanamycin in the same range of concentrations were used as a positive control. The bacterial stock cultures were maintained on nutrient agar plates. A loopful of bacterial cells from the nutrient agar plates was inoculated into 100 mL nutrient broth in 250 mL side arm Erlenmeyer flask and incubated at 37 °C for 16 h with vigorous shaking. After incubation, the culture was diluted with fresh media to give an O.D600 nm of 0.1. Fifty μL of standardized 18 h incubated bacterial culture was introduced into test tubes containing 5 mL media followed by the addition of various concentration of the compounds studied. The MIC was recorded as the lowest concentration that inhibits the growth of the bacterial strains. All assays were performed in triplicate and MIC’s values are given in mg/mL.

## 4. Conclusions

Novel imidazole and benzimidazole compounds with a sulfonamido moiety as substituent, *i.e.*, **3a**–**c**, **4a**–**c**, and **9** in addition to novel bis-sulfonamide compound *i.e.*, **11** were successfully synthesized through simple methods. The structures for the synthesized compounds were confirmed by FTIR, NMR, and HRMS studies. These compounds were evaluated for *in vitro* antibacterial activities against ten strains of bacteria. Compounds **3c**, **9** and **11** demonstrated the highest bioactivities among the compounds, however, most of them showed significant activities for both Gram-positive and Gram-negative bacteria. Such results are encouraging for synthesis of promising new complexes from these compounds with several metals to evaluate their biological activity in the near future. Further studies of these new synthesized sulfonamide derivatives by the same authors are in progress related to their use as ligands and their complexes.

## Supplementary Materials

Supplementary materials can be accessed at: http://www.mdpi.com/1420-3049/18/10/11978/s1.
