# Supplementary material for: Synthesis and Antibacterial Evaluation of Some Novel Imidazole and Benzimidazole Sulfonamides
Source: Molecules. 2013 Sep 26;18(10):11978–95. doi: 10.3390/molecules181011978 (PMC6270528; doi:10.3390/molecules181011978)

Supporting Materials

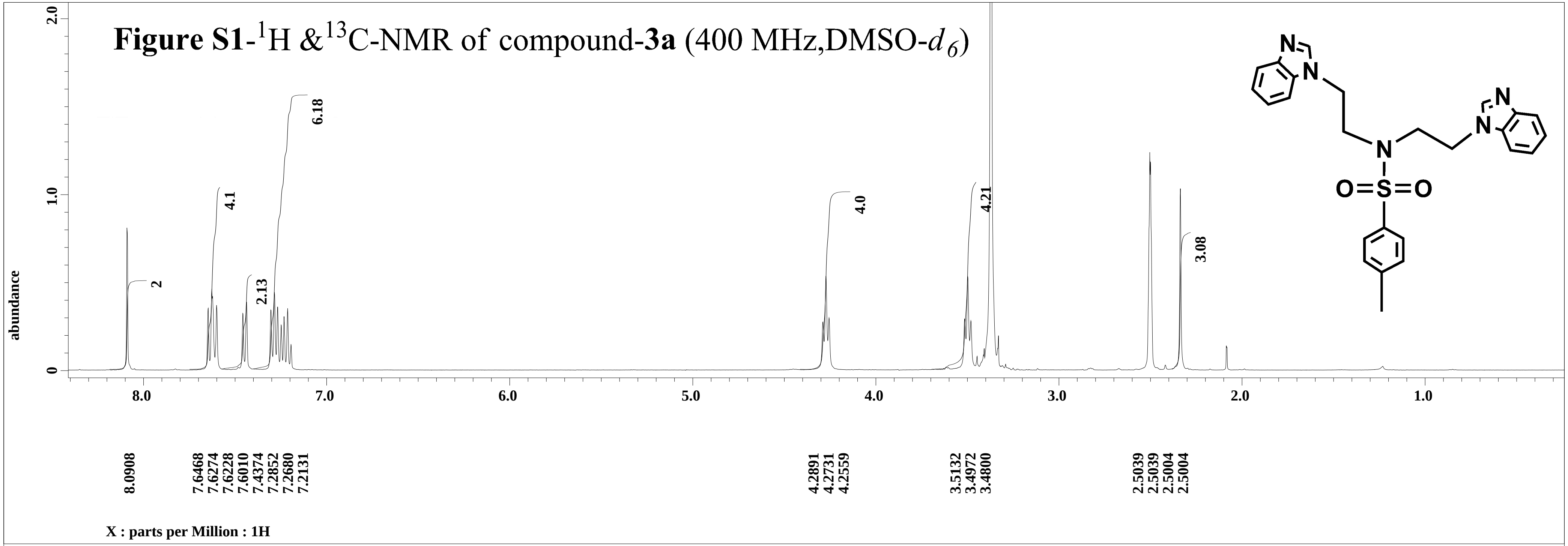

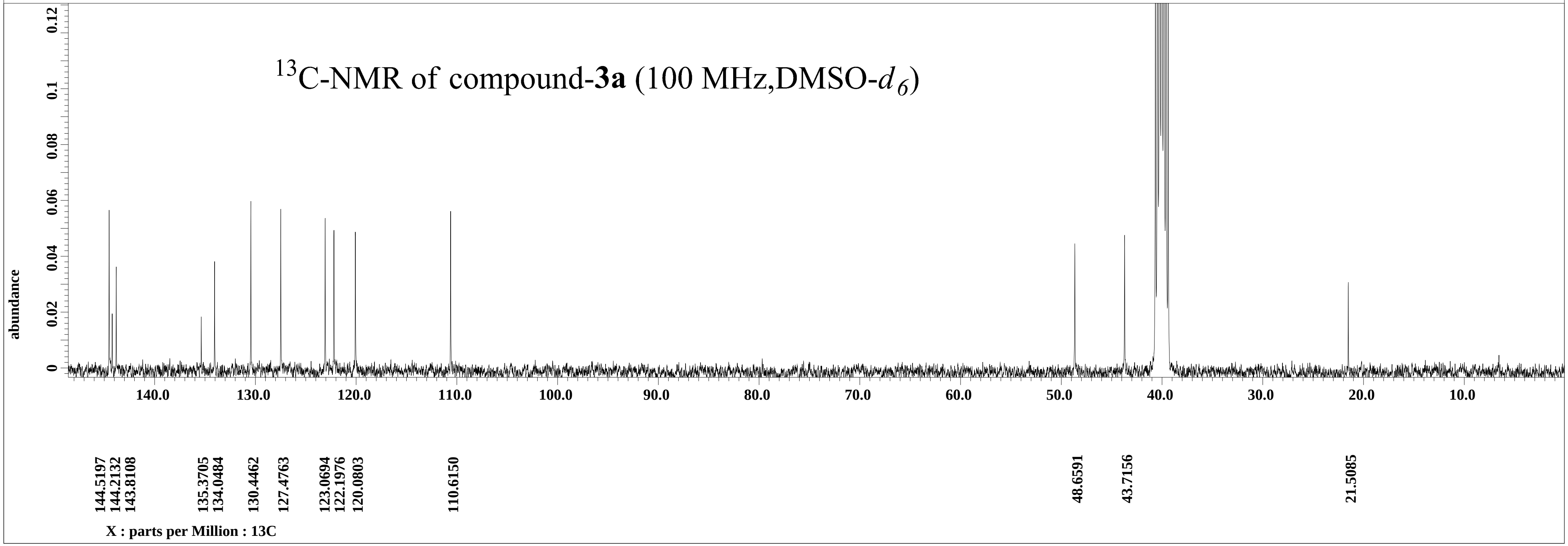

Figure S2-^{13}C-NMR-Pendant of compound-**3a** (100 MHz,DMSO-d_6)

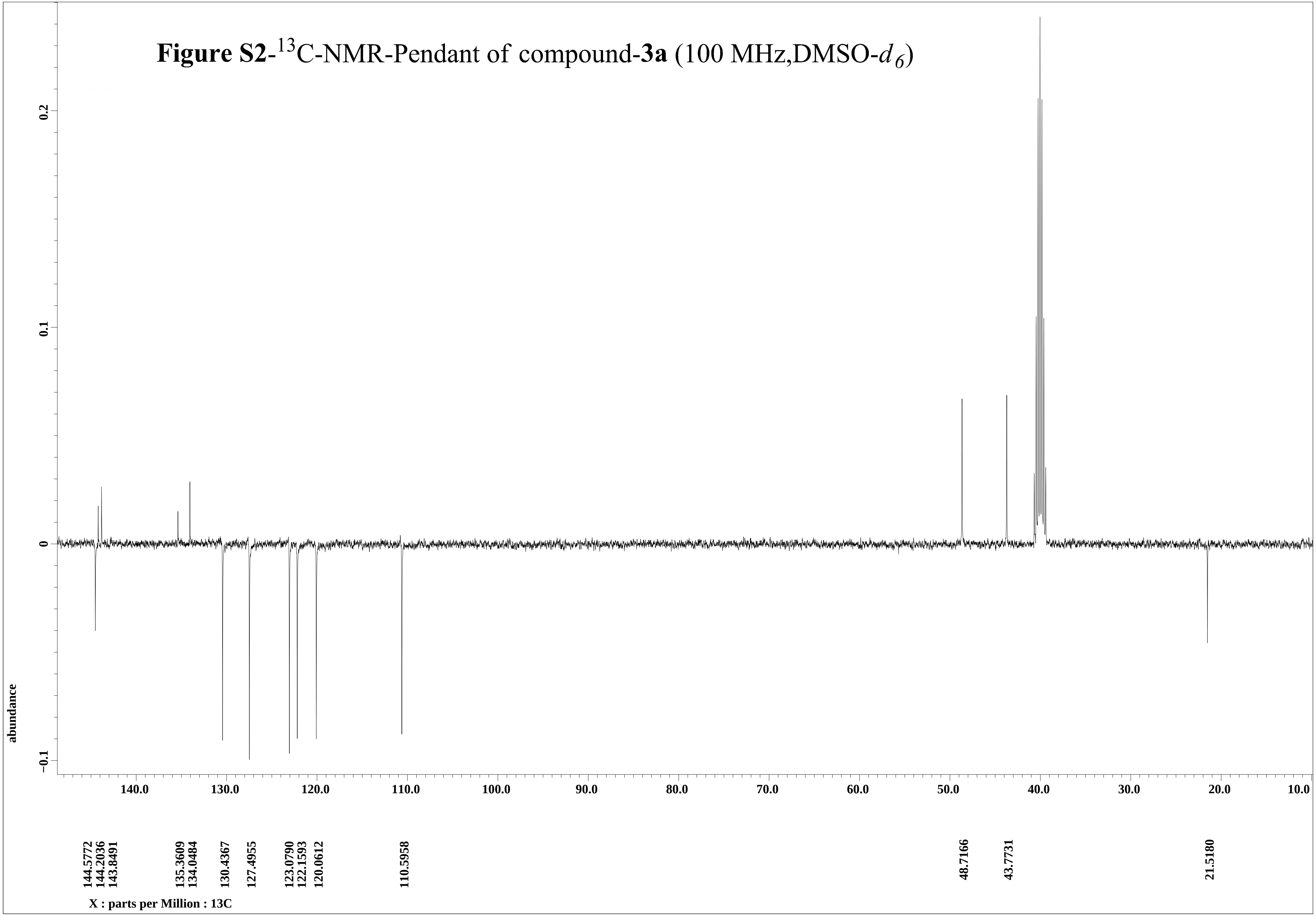

Figure S3-^{1}H &^{13}C-NMR of compound-**3b** (400 MHz,DMSO-d_6)

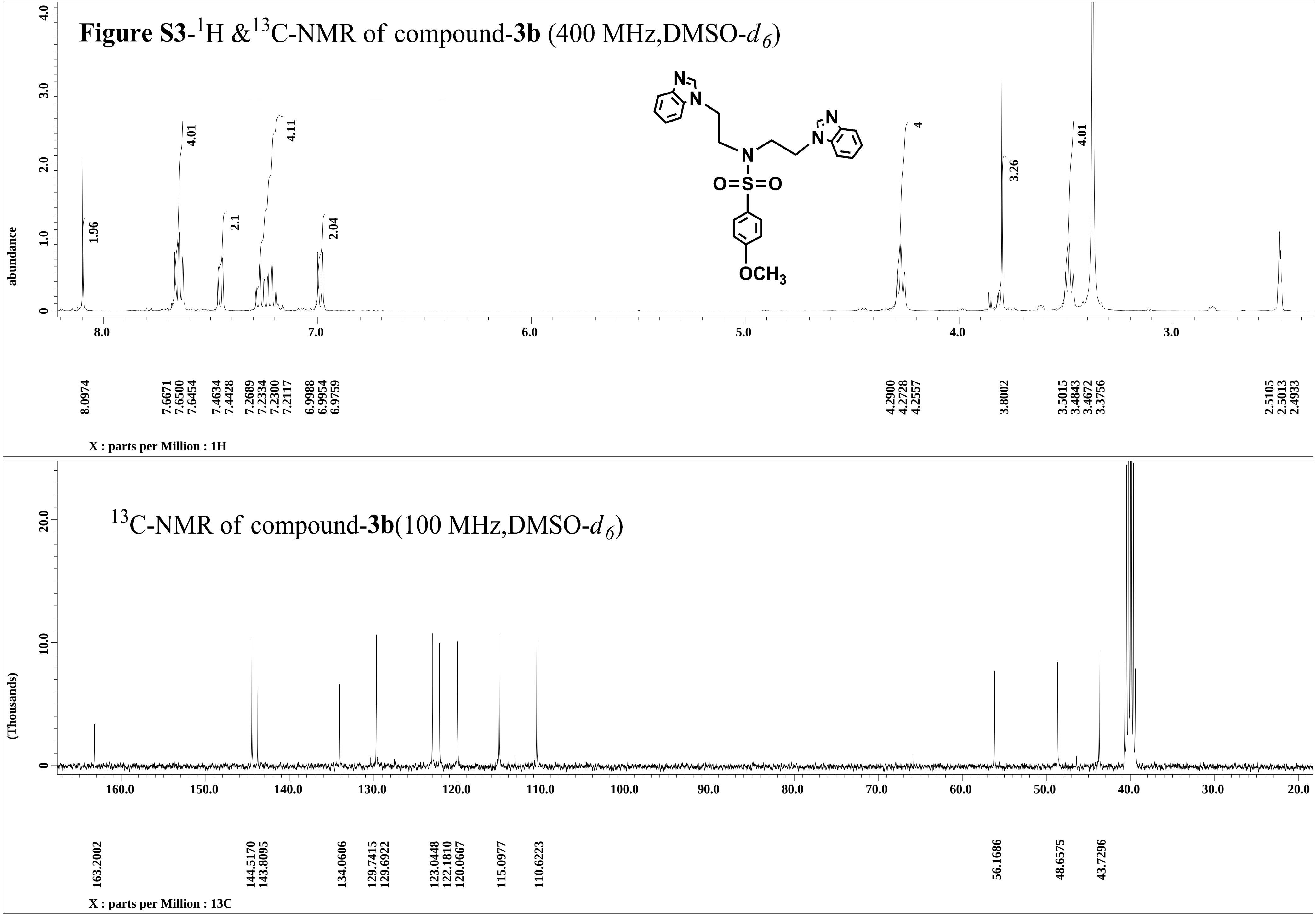

^{13}C-NMR of compound-**3b**(100 MHz,DMSO-d_6)

Figure S4-^{13}C-NMR Pendant of compound-**3b** (100 MHz,DMSO-d_6)

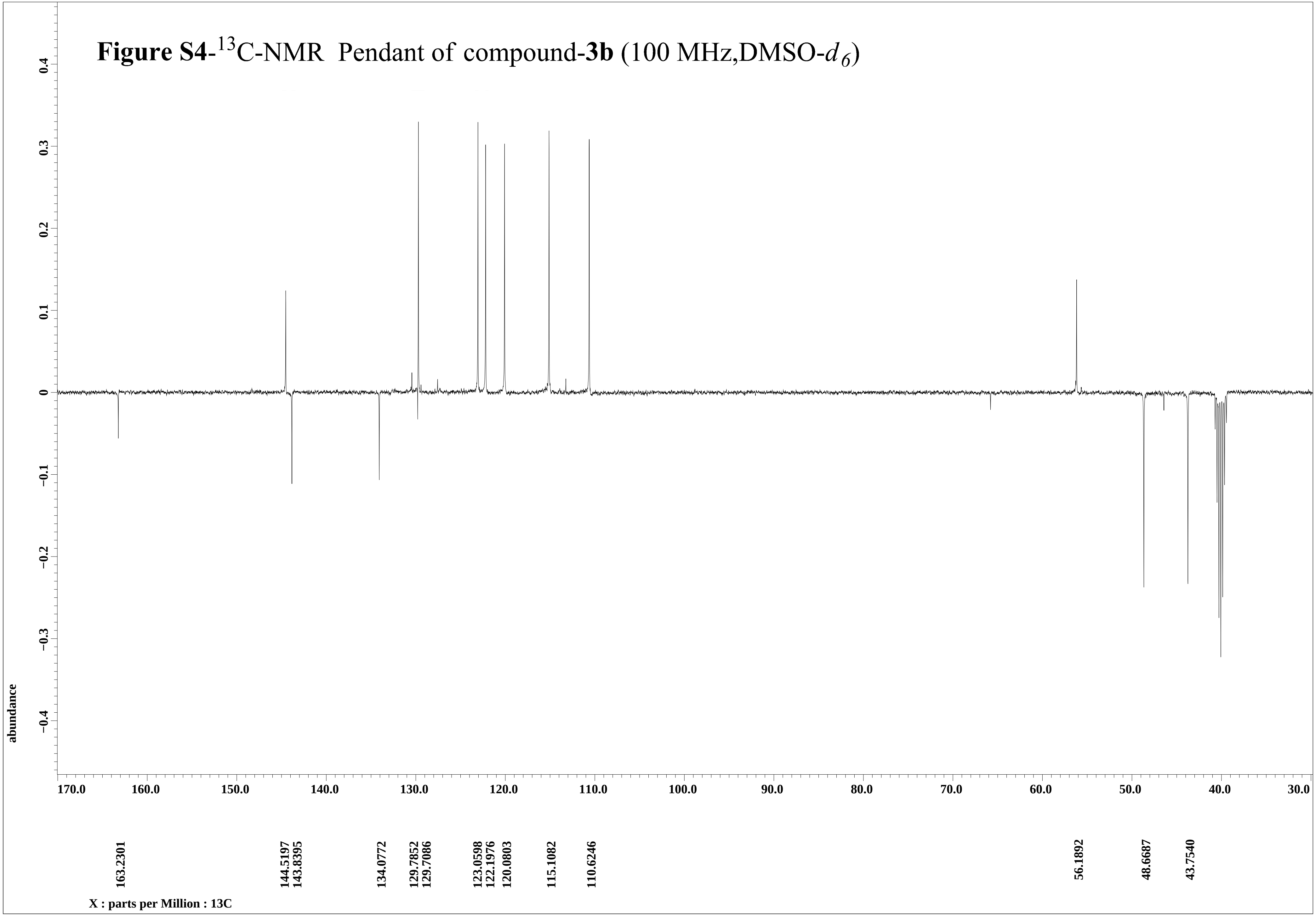

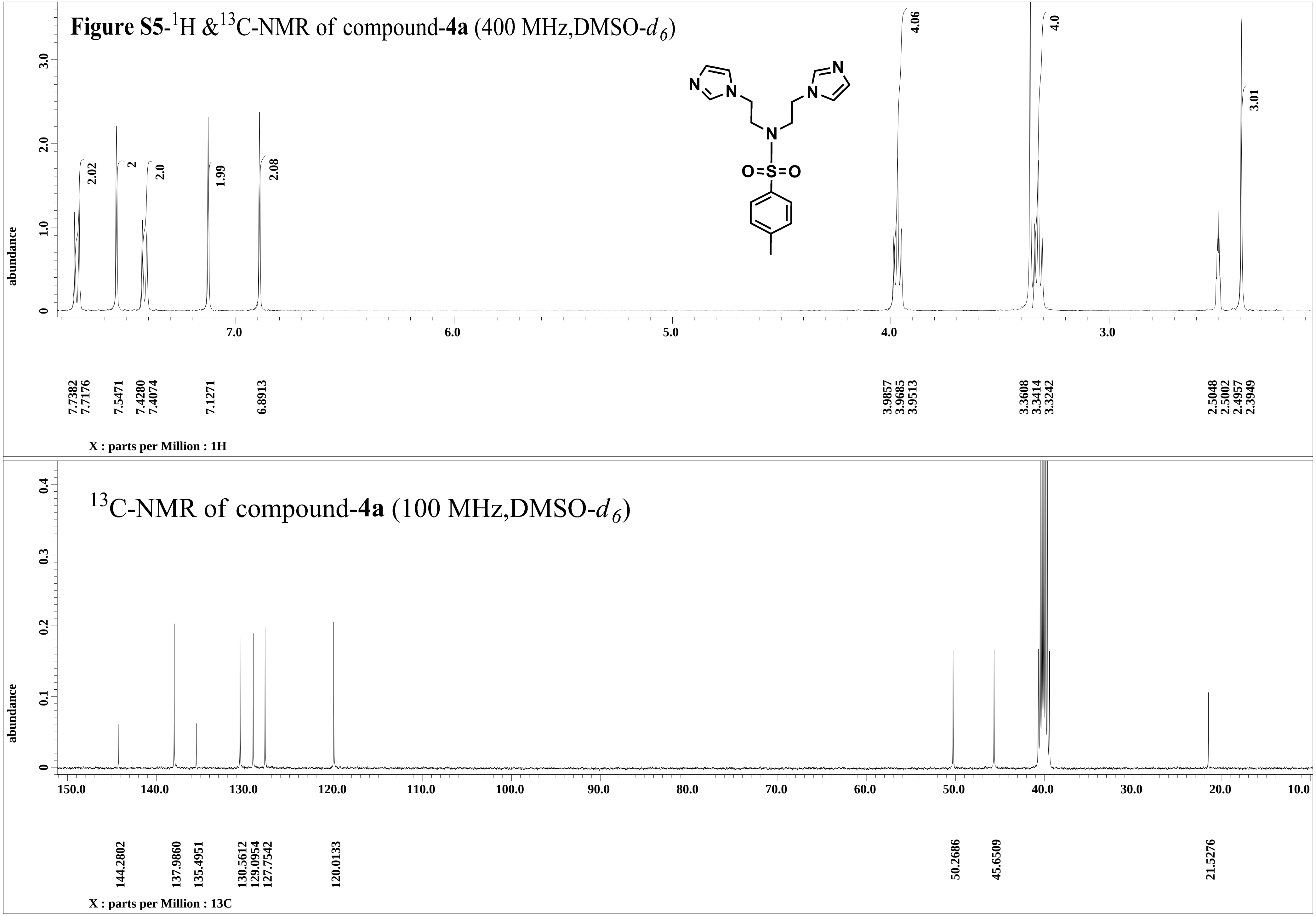
Figure S5-^{1}H &^{13}C-NMR of compound-4a (400 MHz,DMSO-d_6)
abundance
2.02
2
2.0
1.99
2.08
4.06
4.0
3.01
7.7382
7.7176
7.5471
7.4280
7.4074
7.1271
6.8913
3.9857
3.9685
3.9513
3.3608
3.3414
3.3242
2.5048
2.5002
2.4957
2.3949
X : parts per Million : 1H
^{13}C-NMR of compound-4a (100 MHz,DMSO-d_6)
abundance
144.2802
137.9860
135.4951
130.5612
129.0954
127.7542
120.0133
50.2686
45.6509
21.5276
X : parts per Million : 13C

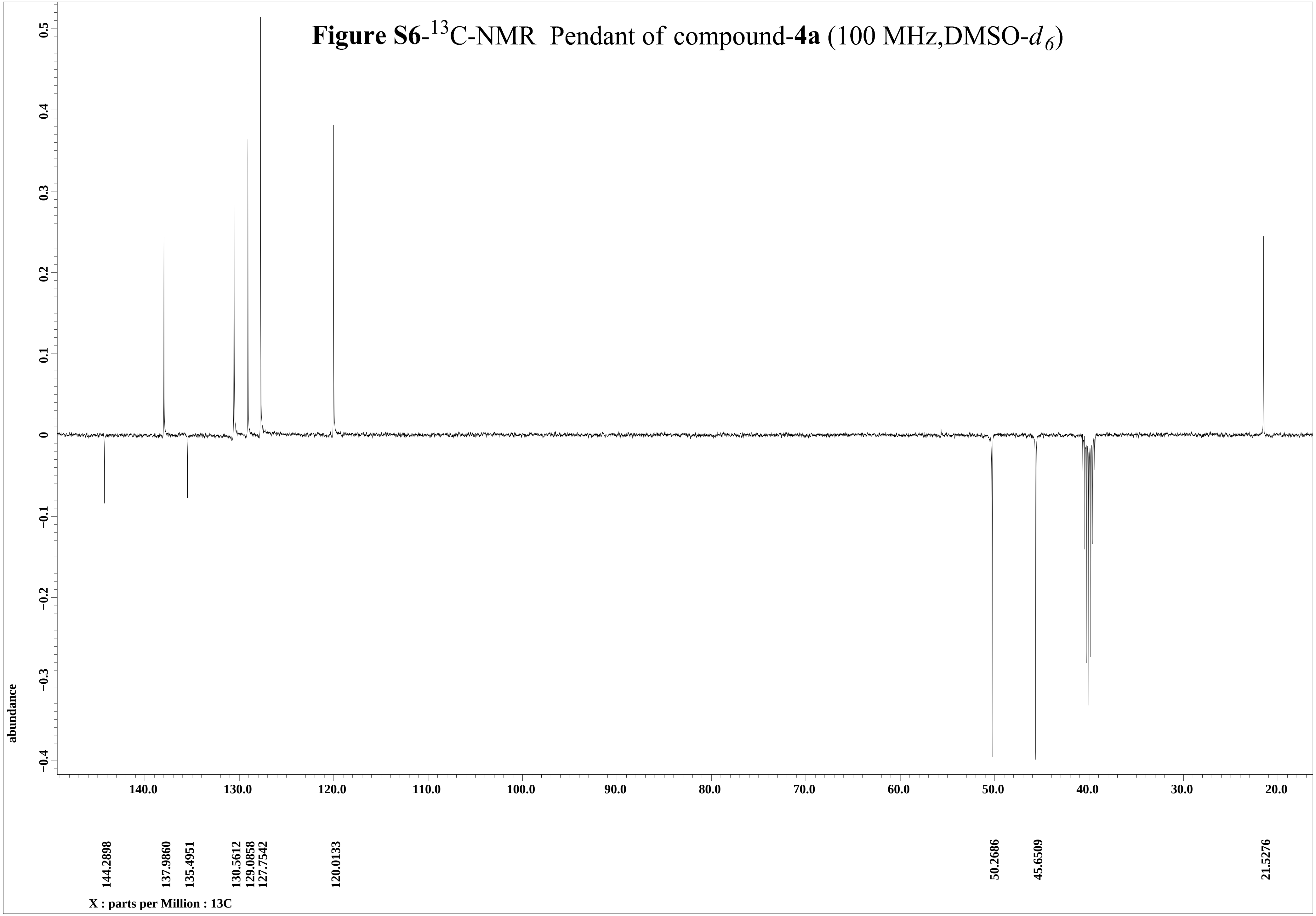

Figure S6-^{13}C-NMR Pendant of compound-**4a** (100 MHz,DMSO-d_6)

Figure S7-^{1}H &^{13}C-NMR of compound-**9** (400 MHz,$CDCl_3$)

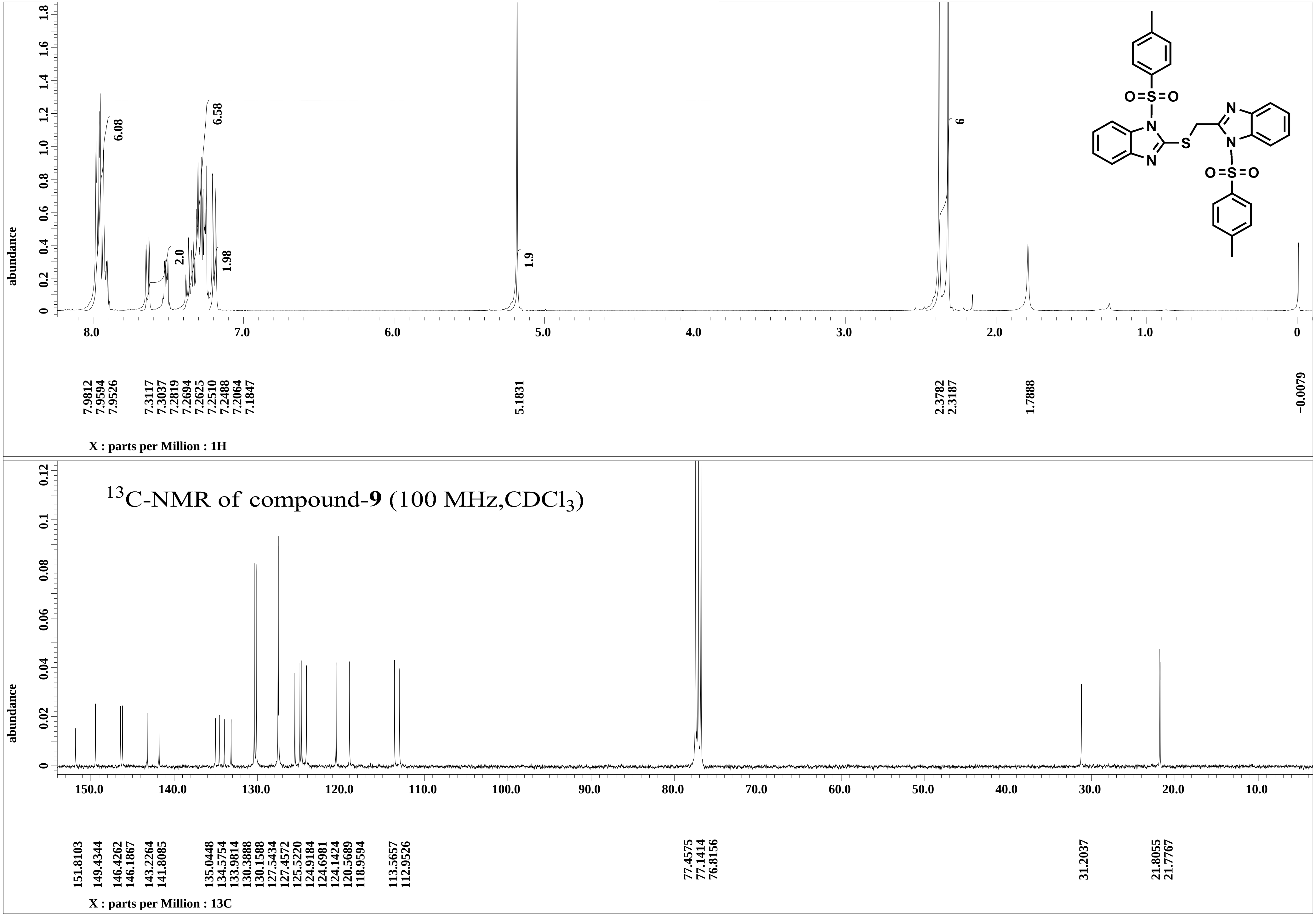

Figure S8-^{1}H &^{13}C-NMR of compound-**11** (400 MHz,$CDCl_3$)

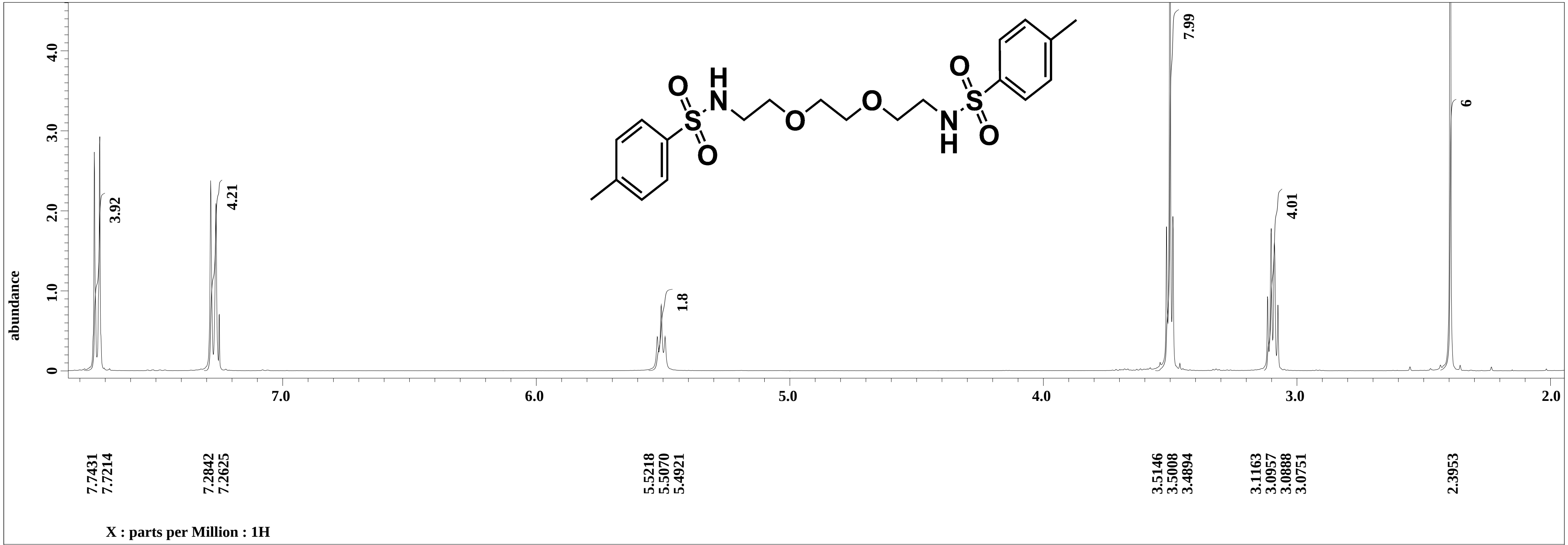

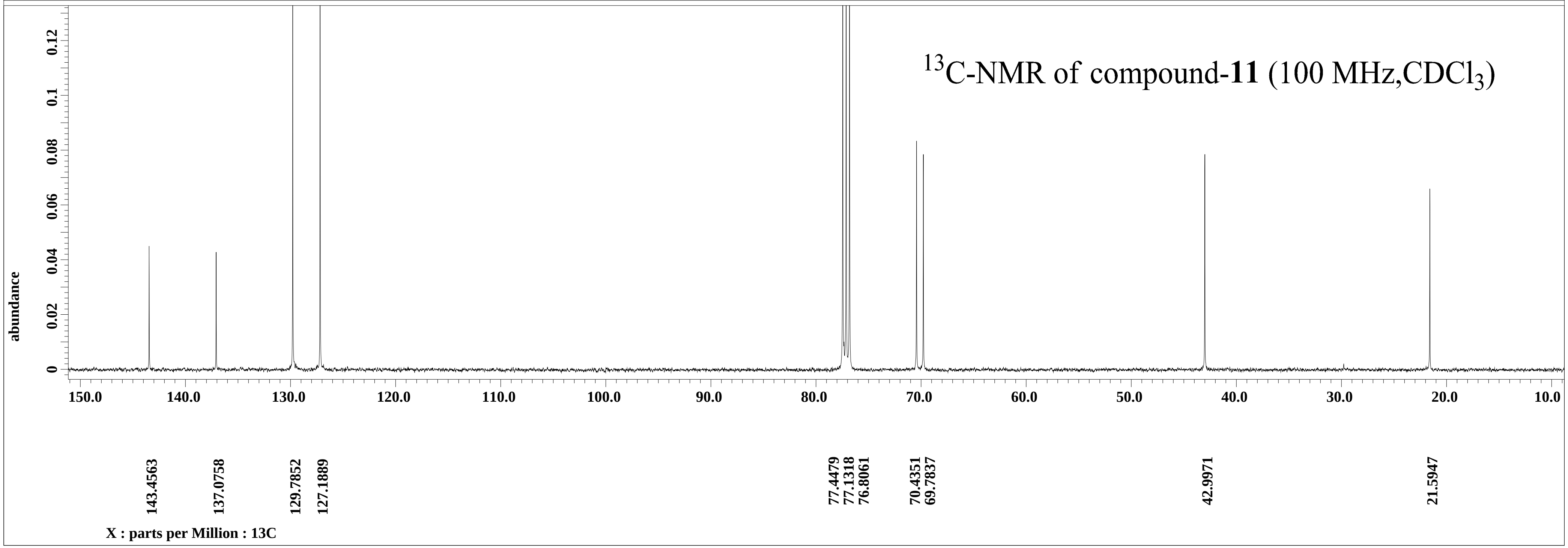

Figure S9- HRMS of compound-**3a**

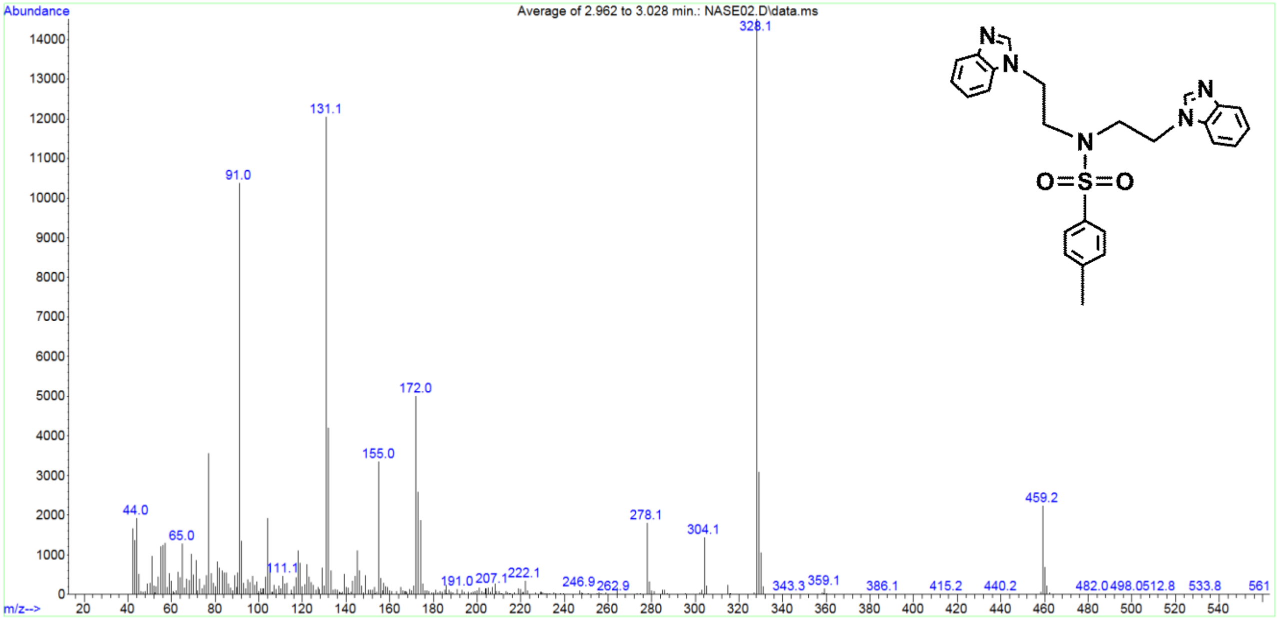

Figure S10- HRMS of compound-**4a**

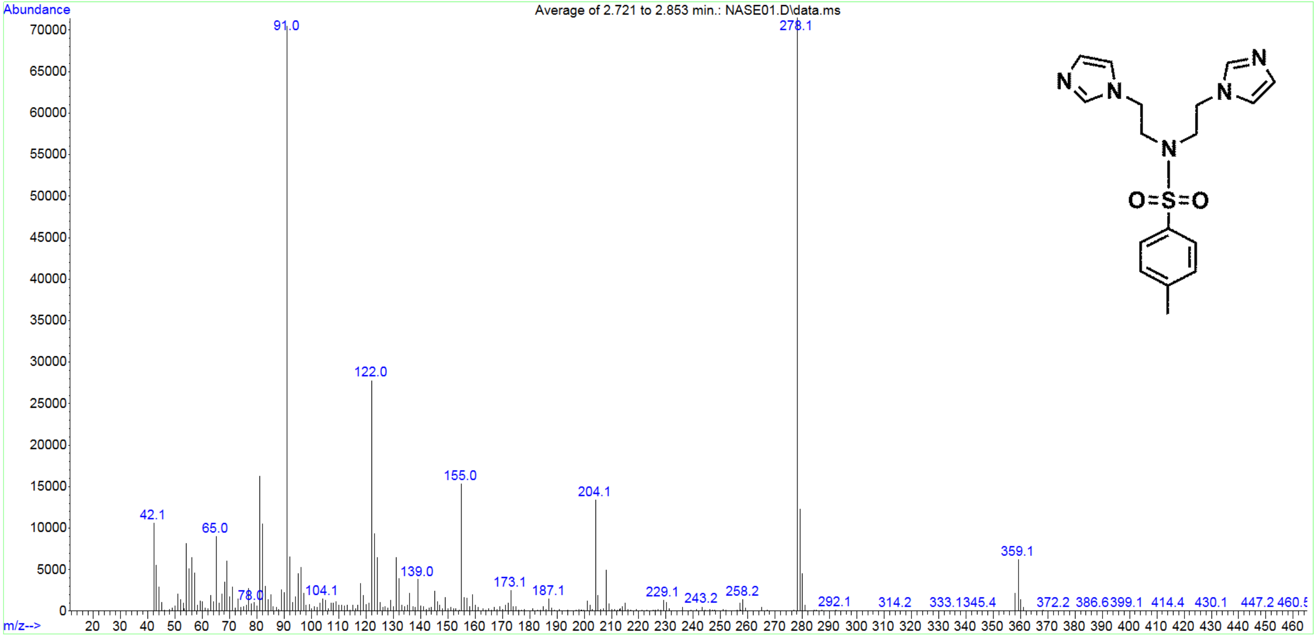

Supplement: Supplementary file 1 [file molecules-18-11978-s001.pdf]
